# A new paradigm evaluating cost per cure of HCV infection in the UK

**DOI:** 10.1186/s41124-016-0002-z

**Published:** 2016-04-14

**Authors:** Stephen T. Barclay, Graham S. Cooke, Elizabeth Holtham, Aline Gauthier, Jeremie Schwarzbard, Petar Atanasov, William L. Irving, On behalf of HCV Research UK

**Affiliations:** 1 Walton Liver Clinic, Glasgow Royal Infirmary, Glasgow, UK; 2 Division of Infectious diseases, Imperial College London, London, UK; 3 NIHR Nottingham Digestive Diseases Biomedical Research Unit, University of Nottingham, Nottingham, UK; 4 Amaris Ltd, London, UK; 5 Microbiology, Queen’s Medical Centre, Nottingham, NG7 2UH UK

**Keywords:** Hepatitis C, Protease inhibitors, Cost, SVR

## Abstract

**Background:**

New interferon (IFN)-free treatments for hepatitis C are more effective, safer but more expensive than current IFN-based therapies. Comparative data of these, versus current first generation protease inhibitors (PI) with regard to costs and treatment outcomes are needed. We investigated the real-world effectiveness, safety and cost per cure of 1st generation PI-based therapies in the UK.

**Methods:**

Medical records review of patients within the HCV Research UK database. Patients had received treatment with telaprevir or boceprevir and pegylated interferon and ribavirin (PR). Data on treatment outcome, healthcare utilisation and adverse events (AEs) requiring intervention were collected and analysed overall and by subgroups. Costs of visits, tests, therapies, adverse events and hospitalisations were estimated at the patient level. Total cost per cure was calculated as total median cost divided by SVR rate.

**Results:**

154 patients from 35 centres were analysed. Overall median total cost per cure was £44,852 (subgroup range,: £35,492 to £107,288). Total treatment costs were accounted for by PI: 68.3 %, PR: 26.3 %, AE management: 5.4 %. Overall SVR was 62.3 % (range 25 % to 86.2 %). 36 % of patients experienced treatment-related AEs requiring intervention, 10 % required treatment-related hospitalisation.

**Conclusions:**

This is the first UK multicentre study of outcomes and costs of PI-based HCV treatments in clinical practice. There was substantial variation in total cost per cure among patient subgroups and high rates of treatment-related discontinuations, AEs and hospitalisations. Real world safety, effectiveness and total cost per cure for the new IFN free combinations should be compared against this baseline.

**Electronic supplementary material:**

The online version of this article (doi:10.1186/s41124-016-0002-z) contains supplementary material, which is available to authorized users.

## Background

Hepatitis C virus (HCV) infection remains a global public health issue with most recent estimates suggesting that more than 180 million people have been infected worldwide [1]. The combination of peginterferon alfa (IFN), ribavirin (RBV) and one of the first generation protease inhibitors (PI), boceprevir (BOC) or telaprevir (TVR) was the standard care for genotype 1 HCV up to 2014 [2, 3].

IFN based HCV treatment is able to cure HCV (as defined by sustained virological response, SVR) [2] which is associated with improved clinical outcomes [4–6]. However, common adverse events (AEs) associated with traditional 1^st^ generation PI-based therapies include skin rashes, pruritus, anaemia, neutropaenia, nausea and dysgeusia [7]. In addition, treatment with IFN may cause psychiatric adverse events such as depression [8]. Evidence suggests that treatment-related AEs have a negative impact on health-related quality of life (HRQoL) leading to higher rates of premature discontinuation and lower SVR rates than reported from clinical trials [9, 10]. Evidence from clinical trials suggests that in treatment naïve genotype 1 patients, triple therapy of PR + 1^st^ generation PIs results in SVR rates of between 65–75 % [11–14] however, lower effectiveness has been reported in real-world studies (44–55 %) [15–18].

The treatment landscape of HCV is changing rapidly with the emergence of novel highly effective all oral combination therapies active against genotype 1 [2]. With better efficacy, improved safety and substantially shortened treatment durations, these IFN – and RBV-free treatments are expected to become the cornerstone of HCV therapy, [19–23]; these advances represent a major treatment improvement for patients. However, current pricing, combined with the likely increased patient demand, means these regimens pose a significant budgetary impact in developed healthcare systems [24]. Recently, the concept of “cost per SVR” has evolved to take account of opportunity costs (i.e. the loss of potential gain from other alternatives when one alternative is chosen) in HCV management. More data is required to understand the total cost per SVR of existing IFN-based therapies to allow meaningful comparison with newer interferon free regimens.

To date there have been no published real-world studies assessing the cost per cure, safety and effectiveness of PR + 1^st^ generation PI triple therapy in the UK. This study aimed to estimate the total cost per HCV patient cured associated with first generation PI-based therapy in a real world setting, across a large number of centres. As secondary objectives, this study also aimed at estimating the effectiveness, safety and tolerability of these regimens.

## Methods

### Study design and patients

This was a retrospective observational multi-centre study of patients enrolled in the HCV Research UK biobank, who received a first generation PI-based regimen between July 2012 and July 2014 through National Health Service (NHS) hospitals following recommendation by the National Institute for Health and Care Excellence (NICE) [25, 26] and the Scottish Medicines Consortium. HCV Research UK is a cohort of 10,000 patients, plus database and biorepository, enrolled with informed consent from 59 centres across England, Scotland and Wales [27]. Patients within HCV Research UK were considered eligible for this analysis if they were 18 years of age or older, had HCV genotype 1 infection and had received either TVR or BOC in association with PEG + RBV with a known treatment outcome. Patients were randomly selected for this study from all eligible patients using a number generator. A TVR: BOC sample ratio of 2:1 was chosen to reflect use in clinical practice among the HCV Research UK cohort.

### Data collection

Baseline data available from within the HCV Research UK database were supplemented by a detailed clinic case note review. Data collected included patient characteristics at initiation of the PI-based treatment (age, gender, ethnicity, liver disease status, genotype, blood test results, comorbidities recorded in the HCV Research UK database relating to renal failure, diabetes, HIV co-infection, depression, haemophilia and cancer (other than HCC)), previous HCV therapy (type of treatment and associated response), details regarding the PI-based therapy, treatment outcome, safety and healthcare resource use (nurse and physician visits, tests and investigations, medications used to treat AEs, and hospitalisations). Details on comorbidities at baseline were gathered through a review of the patient’s clinical notes as well as patient questionnaire. IL28B genotyping at locus rs12979860 was performed (by Micropathology Ltd, Coventry, UK) post-hoc using a Roche Hybprobe kit and meltcurve analysis.

### Assessment of outcomes

The outcomes of interest included SVR, which was assessed at 12 or 24 weeks. The incidence and type of adverse events (AEs) requiring therapeutic intervention, healthcare resource use, treatment discontinuation and/or hospitalisation was collected by the study centre through a standardised questionnaire and quality controlled by a study coordinator. The relationship of HCV therapy to each AE was assessed independently by two physicians (STB, GSC), and categorised as “likely”, “unlikely” or “can’t tell”. Any discrepancy between assessments was resolved by discussion. In total, there were 12 discrepancies after the initial individual assessments, all of which were agreed on after discussion with no further action needed.

Costs were estimated from the NHS perspective. The unit costs to be applied were derived from the British National Formulary for drugs [28]. The cost of managing HCV was estimated by applying the national unit costs to the number of physician and nurse visits, medications and tests [29]. The cost of hospitalisations was estimated based on Health Resource Group (HRG) codes classifying the reasons for hospitalisation and procedures undertaken during the stay [30]. Corresponding unit costs were obtained from the NHS reference costs [31]. Total costs were estimated and cost per cure was calculated as total median cost divided by SVR rate. For the conversion of UK pound (£) to US dollar ($), the Bank of England exchange rate was applied (£1 = $1.4905) [32].

### Statistical analysis

Sample size was not formally assessed and the number of patients enrolled was based on time and resource constraints. Descriptive statistics were analysed according to treatment status (naïve vs experienced) and liver disease status (cirrhosis vs no cirrhosis) at baseline. The proportion of patients achieving SVR was determined overall, by treatment (BOC vs TVR) and by liver disease status. The calculation of SVR took into account all patients, including those who discontinued therapy prematurely. The statistical significance was tested for each comparison using a Chi-square test.

Logistic regression was conducted to assess the impact of patient characteristics on the probability of reaching SVR. The list of covariates was specified a priori and included age, liver disease status, gender, treatment naïve vs experienced, HIV co-infection, IL28B subtypes, history of depression, diabetes, genotype and race. A stepwise procedure was run to identify the most significant covariates.

The incidence of AEs and of AEs assessed as “likely” to be related to HCV treatment was estimated overall and by type of AE. The incidence of AEs leading to treatment discontinuation and/or to hospitalisation was also estimated.

To identify factors which have an impact on the probability of experiencing an AE, a logistic regression was conducted. The list of covariates included age, gender, treatment regimen, past treatment history, liver disease status, HIV status, history of depression and diabetes.

Sensitivity analyses were performed to assess the impact of PI drug price discounts of 30 %, 40 % and 50 % and the impact of including management costs of ‘can’t tell’ AEs on the total cost per cure.

## Results

### Baseline characteristics

From the initial 160 patients selected for this study from the HCV Research UK database, 8 patients did not in fact start TVR/BOC therapy, 3 patients were switched from one PI to the other, 1 patient had a genotype 3 infection, and for 5 patients, the clinics concerned notified us that they would not be able to retrieve and return the data within our specified timeframe, resulting in a total of 17 patients being excluded. An additional 11 patients were randomly selected, resulting in a total of 154 patients entered into this study derived from 35 centres.

Table 1 shows the baseline characteristics of all patients enrolled in this study. Of all patients, 63/154 (40.9 %) had at least one comorbidity and 8/154 (5.2 %) had at least two. The most common comorbidities at baseline were reported history of depression (40/154, 26 %), HIV co-infection (10/154, 6.5 %), diabetes (8/154, 5.2 %), haemophilia (8/154, 5.2 %), cancer (6/154, 3.9 %) and renal failure (1/154, 0.7 %). Over half the patients were treatment experienced (80/154, 52 %), the majority having been treated with PEG + RBV (62/80, 77.5 %). The nature of the previous treatment regimen was not available for the other 18 patients. Among treatment-experienced patients, 35/80 (43.7 %) were relapsers and 36/80 (45.0 %) were non-responders. This information was not available for 9 patients.

**Table 1:** Baseline characteristics for total cohort

	All patients (*n* = 154)
Age [Mean, (SD)]	49.6 (9.01)
Gender	
Male [%]	73.4 %
Female [%]	26.6 %
Ethnicity	
White [%]	94.2 %
Liver disease (Metavir stage)	
Non-cirrhotic (F0-F3) [%]	78.6 %
Cirrhotic (F4 including 1 HCC) [%]	21.4 %
Treatment history	
Experienced [%]	52.0 %
Naïve [%]	48.0 %
Response to previous therapy	
Relapse [%]	43.7 %
No response [%]	45.0 %
Missing [%]	11.3 %
HCV genotype	
Genotype 1a [%]	51.3 %
Genotype 1b [%]	18.8 %
G1 subtype Missing [%]	29.9 %
HCV therapy	
Telaprevir treatment [%]	69.1 %
Boceprevir treatment [%]	33.1 %
IL28B status (SNP rs12979860)	
Heterozygous CT [%]	56.9 %
Homozygous CC [%]	29.4 %
Homozygous TT [%]	13.7 %
Missing [%]	0.7 %
Comorbidities	
Depression [%]	26.0 %
HIV co-infection [%]	6.5 %
Diabetes [%]	5.2 %
Haemophilia [%]	5.2 %
Cancer [%]	3.9 %
Renal failure [%]	0.7 %

More treatment-experienced patients had cirrhosis compared to treatment-naïve (24/80 (30.0 %) vs 9/74 (12.2 %); *p* = 0.007). Patients with cirrhosis were significantly older than those with milder liver disease (stages F0 – F3) (55.6 years (SD: 7.16) vs 48.1 years (SD: 8.9);*p* < 0.0001).

### Effectiveness results

Overall 96/154 (62.3 %) patients achieved SVR. SVR rate in patients treated with BOC was 63.1 % (65/103) and 60.8 % (31/51) in patients treated with TVR (*p* = 0.89). The SVR rate among patients who were treatment-naïve was 51/74 (68.9 %) compared to 12/36 prior non- responders (33.3 %; *p* = 0.004, Table 2). Patients with cirrhosis showed consistently lower SVR compared to those with milder liver disease regardless of previous treatment status. The logistic regression found age, cirrhotic status and IL28b genotype to be significant predictors of SVR (Table 3). Patients with cirrhosis were almost 3 times less likely to achieve SVR compared to those with milder liver disease (F0 – F3) (OR = 0.35; 95 %CI: 0.13, 0.94). Patients with a CC genotype of IL28b were around 9 times more likely to achieve SVR than those with non-CC genotype (OR = 0.116; 95 %CI: 0.037, 0.365).

**Table 2:** SVR rate by regimen, cirrhosis status and prior treatment history

Total (*n* = 145^**a**^)	Treatment-naïve patients (*n* = 74)	Prior relapsers (*n* = 35)	Prior non-responders (*n* = 36)
SVR rate	68.9 % [57.1;79.2]	82.9 % [66.4;93.4]	33.3 % [18.6;51.0]
By regimen			
BOC triple therapy (*n* = 48)	66.7 % [47.2;82.7] (*n* = 30)	75.0 % [34.9;96.8] (*n* = 8)	30.0 % [6.7;65.2] (*n* = 10)
TVR triple therapy (*n* = 97)	70.4 % [54.8;83.2] (*n* = 44)	85.2 % [66.3;95.8] (*n* = 27)	34.6 % [17.2;55.7] (*n* = 26)
By liver disease stage			
No-cirrhosis (n = 114)	73.8 % [61.5;84.0] (*n* = 65)	86.2 % [68.3;96.1] (*n* = 29)	40.0 % [19.1;64.0] (*n* = 20)
Cirrhosis or HCC (*n* = 31)	33.3 % [7.5;70.1] (*n* = 9)	66.7 % [22.3;95.7] (*n* = 6)	25.0 % [7.3;52.4] (*n* = 16)

^a^ Previous treatment outcome status was not available for 9 patients

**Table 3:** Logistic regression: Probability of achieving SVR

Parameter	OR	95 % Wald Confidence Limits		Standard Error	*P* value
Age	0.94	0.90	0.99	0.03	0.02
Gender	1.20	0.49	2.93	0.23	0.69
Female (vs male)					
Liver disease	0.35	0.13	0.94	0.25	0.04
Cirrhosis (vs no cirrhosis)					
Treatment status	0.84	0.35	1.99	0.22	0.69
Experienced (vs naïve)					
HIV co-infection (*n* = 10)	2.56	0.44	14.77	0.45	0.29
Absence (vs presence)					
Depression (*n* = 40)	0.88	0.35	2.22	0.24	0.79
Absence (vs presence)					
Diabetes (*n* = 8)	4.94	0.62	39.56	0.53	0.13
Absence (vs presence)					
IL28b	0.12	0.04	0.37	0.29	0.0002
Non-CC (vs CC)					
Genotype	1.09	0.37	3.22	0.29	0.28
G1a (vs G1b)					
Race	0.31	0.04	2.42	0.52	0.27
Other (vs white)					

### Safety analysis

Over the study period 70/154 (45 %) of the study population experienced at least one AE requiring intervention. The total number of AEs requiring intervention was 178 (see Additional file 1 for details). 141/178 (79.2 %) of these were assessed as “likely” to be related to treatment. Twenty-two of the 178 (12.4 %) AEs requiring intervention were due to anaemia. This was managed by ribavirin dose reduction (16/22, 72.7 %), treatment with erythropoietin analogues (6/22, 27.3 %) and/or blood transfusion (15/22, 68.2 %). All the patients who required intervention for neutropaenia, (*n* = 6) were treated with GCSF, and the single case of thrombocytopenia (*n* = 1) was managed with a dose reduction.

Fifty-five out of 154 (33 %) patients stopped therapy prematurely – of those 17/55 (30.9 %) because of adverse events, 24/55 (43.6 %) patients failed stopping rules, 12/55 (21.8) patients withdrew themselves from therapy, and for 2/55 (3.6 %) patients the physician withdrew therapy. There were 36 hospitalisations amongst 29 patients and, of those, 16 (44.4 %) hospitalisations were classified as “likely” to be treatment related, comprising of 15 hospitalisations due to anaemia (all were associated with blood transfusion) and 1 due to severe headache (see Supplementary material).

Using logistic regression, liver disease status, treatment regimen, gender and past treatment experience were statistically significant predictors of the incidence of any AE (see Supporting material). Cirrhotic patients had a 3-fold increased risk of experiencing an AE compared with patients with no cirrhosis (*p* = 0.0206). Patients on TVR were twice as likely to experience an AE compared to patients on BOC (*p* = 0.0445). Females had approximately a 2-fold increased risk of AEs compared to males (*p* = 0.0384) as did treatment-naïve patients compared to experienced patients (*p* = 0.0476).

### Economic assessment

The overall median total cost per HCV cure was £44,852 ($66,852). Median cost per HCV cure varied according to prior treatment status: £83,948 ($125,125) for previous non-responders, £39,150 ($58,353) for prior relapsers and £37,958 ($56,576) for treatment naïve patients (Table 4).

**Table 4:** Cost per HCV cure – by prior treatment and cirrhotic status

Total (*n* = 154)	Treatment naïve patients (*n* = 74)	Prior relapsers (*n* = 35)	Prior non-responders (*n* = 36)	Prior treatment status unknown (*n* = 9)
Overall	£ 37,958	£ 39,150	£ 83,948	£ 61, 804
By liver disease stage				
Non cirrhotic (*n* = 114)	£ 35,492	£ 37,455	£ 73,098	£ 49,070
Cirrhotic (*n* = 31)	£ 61,496	£ 49,016	£ 107,288	NA

Patients with cirrhosis consistently showed higher costs per cure compared to those with milder liver disease across subgroups - £61,496 ($91,660) vs £35,492 ($52,900) in treatment naïve patients; £49,016 ($73,058) vs £37,455 ($55,827) in prior relapsers; £107,288 ($159,913) vs £73,098 ($108,953) in non-responders.

Using NHS list prices for the base case analysis total direct cost associated with the management of the 154 HCV patients was estimated to be £4,289,909 ($6,394,109). The total drug costs for the 154 patients associated with PI therapies accounted for 68.3 % of total cost (£2,930,769; $4,368,311), PEG accounted for 19.5 % (£834,485; $1,243,800) and RBV, 6.8 % (£290,975; $433,698) (Table 5). In the base case analysis, ‘other’ costs accounted for 5.4 % of total costs (£233,780; $348,449), comprising of consultant/nurses visits (59.9 %; £139,992); tests (22.5 %; £52,616); medications for adverse events (including blood transfusions) (7.4 %; £17,233); other investigations (6.3 %; £14,576) and hospitalisations (3.9 %; £9,209) (Table 5). Management of depression accounted for a small fraction of these costs.

**Table 5:** Total costs

Costs	Base case Total cost (in £)	%	30 % discount on PI	%	40 % discount on PI	%	50 % discount on PI	%
PIs	2,930,769	68.3	2,051,538	60.2	1,758,461	56.4	1,465,385	51.9
PegINF	834,485	19.5	834,485	24.5	834,485	26.8	834,485	29.6
Ribavirin	290,975	6.8	290,975	8.5	290,975	9.3	290,975	10.3
Physician visits	139,992	5.4	232,573	6.8	232,573	7.5	232,573	8.2
Tests	52,616							
Other investigation	14,576							
Hospitalisation	9,209							
Adverse events	17,233							
Depression & hypnotics	54							
Total	4,289,909	100.0	3,409,571	100.0	3,116,494	100.0	2,823,418	100.0
Total median cost per cure (£)	44,852	-	34,416	-	30,968	-	27,233	-

All but 1 (13/14) patient experiencing a ‘likely’ treatment-related hospitalisation had a single hospital episode with 1 patient being admitted twice. The mean number of hospital days spent per hospitalisation was 3.5.

In the UK, treatment is generally supervised by a clinical nurse specialist, however 78/154 (51 %) of patients had at least one consultant (hepatology or infectious diseases) visit and 17/154 (11 %) patients required at least one dermatologist visit during treatment. The mean number of consultant visits per patient during the follow-up was 1.2, with a mean number of nurse visits of 14.5. During the follow-up period, patients had, on average, 13 liver function tests, 10 electrolyte tests and 5 HCV viral load tests. In total ‘other healthcare costs’ accounted for approximately £1,500 per patient; £232,573 per 154 patients.

The PI price sensitivity analysis (30 %, 40 %, 50 % discount) indicated that a 10 % drop in PI drug price was associated with a decrease of 7 % in the cost per cure (Table 5). Discount rates sensitivity analyses stratified according to outcome of previous therapy as well as liver disease status showed clearly that no response to previous therapy as well as the presence of cirrhosis is associated with increases in total costs per cure (see Supporting material).

In the base case analysis, only ‘likely’ hospitalisations and AEs were included. 141 AEs were deemed ‘likely’ and 24 ‘can’t tell’ (the remaining 13 being deemed “unlikely”). The impact of inclusion of cost for ‘can’t tell’ classified AEs in the sensitivity analysis had only a negligible impact on total median cost per SVR - £38,027 vs £37,958 with and without respectively.

## Discussion

The fact that HCV infection will incur a significant future healthcare burden is now commonly accepted. Use of highly effective therapies has been proven to improve SVR rates and therefore reduce HCV related morbidity and mortality [33]. The first IFN-free combinations of directly acting antivirals (DAAs) against hepatitis C (HCV) have been licensed and more are likely to follow in the coming years. These treatments are significantly shorter in duration, yield higher SVR and have fewer AEs [34] than traditional IFN-based therapies. With expected high costs of therapy, it is important to evaluate the cost per SVR in order to maximise efficient use of scarce healthcare resources.

We set out to describe the effectiveness, safety and costs per cure for first generation protease inhibitor based treatment of genotype 1 hepatitis C (the most common genotype), in a real-world UK setting.

Differences between SVR results achieved in trials (efficacy) and those in clinical practice (effectiveness) with PR + 1^st^ generation PIs have been previously documented [11–18]. Such differences have been attributed to the restrictive inclusion criteria of clinical trials with respect to certain groups of patients including those with advanced disease, cirrhosis and high co-morbidity burden.

While we found an overall SVR of 63 % in our sample, comparable with registration trials, there was (as expected) significant variation, with cirrhotic patients and prior non-responders having worse SVR outcomes.

Just over a third (35.7 %) of patients did not complete treatment. The discontinuation rate due to treatment related AE amongst our sample was 11 %. Nearly half of patients in our sample experienced an AE requiring an intervention with 79 % of those AE considered ‘likely’ to be treatment-related. Almost 10 % of all patients required hospitalisation ‘likely’- related to treatment.

Overall median total cost per cure for our sample in the base case analysis was £44,852; however, there was substantial variation among subgroups ranging from £35,492 in treatment naive non-cirrhotics to £107,288 in cirrhotic prior non-responders. In the base case analysis for 154 patients, HCV triple therapy costs accounted for 94.6 % of the total cost (£4,056,229).

The characteristics of our UK cohort and the overall SVR rates are very similar to a study carried out in Germany, although the costs per cure estimated here were significantly lower, a difference driven to a large extent by the higher costs attributed to antivirals in Germany (see Table 6). A recent US study of similar size found a lower overall SVR rate, reflecting in part a slightly older population and a higher proportion of African-Americans, a group where a higher prevalence of IL28b T allele has been associated with poorer treatment outcomes. Despite these differences, cost per cure in the US was substantially higher, likely due to sample differences (e.g. proportion of high cost prior non-responders 46 % US: 22 % UK) as well as differences in clinical AE management (e.g. anaemia) and drug costs).

**Table 6:** Comparison with recent US and German studies

Patients characteristics	Current study (*n* = 154)	Stahmeyer et al. (GER) [35] (*n* = 858)*	Bichoupan et al. (US) [17] (*n* = 147)
Age, years [Mean, (SD)]	49.6 (9.0)	49.2 (11.2)	56 (IQR = 51–61)
Gender, Male [%]	73.4	58	68
Ethnicity, %			
Caucasian [%]	94.20 %	97.30 %	Not reported
Asian [%]	2.70 %	0.90 %	Not reported
African [%]	1.30 %	0.80 %	19 %
Hispanic [%]	Not reported	0.50 %	Not reported
Other [%]	1.80 %	Not reported	Not reported
SVR rate by therapy			
BOC [%]	60.8	58.1	Not reported
TVR [%]	63.1	68.4	44
Cirrhosis [%]	21.40 %	Not reported	36 %
Prior non-responders [%]	22.10 %	Not reported	46 %
Cost drivers			
Drug costs	94.60 %	94.6–97.7 %	89 %
PIs	68.30 %	Not reported	Not reported
TVR	Not reported	Not reported	61
PegIFN	19.50 %	Not reported	24 %
RBV	6.80 %	Not reported	4 %
Outpatient and inpatient care (incl AEs & follow-up)	5.40 %	5.4-3.3 %	8 %
Cost per SVR ($)			
Treatment naïve	56,576	73,473–82,756	Not reported
Relapsers	58,353	78,529–93,758	Not reported
Non-responders	125,125	127,1049–132,204	Not reported
Overall	Not reported	Not reported	189,000

*Costs from Stahmeyer et al. were converted from euros to US dollars (conversion rate: €1 = $1.0985 based on European Central Bank exchange rates, March 2015)

The strength of this study is that it represents a large randomly drawn sample, representing 35 UK centres treating hepatitis C, taken from a large UK database (HCV Research UK) and is thus likely to be representative of overall UK practice. The overall case-mix of patients considered for treatment in coming years is likely to change and the breakdown of outcomes presented according to sub-groups allows comparative analysis for different patient groups.

There are a number of factors which may underestimate the ‘true’ total cost per cure in our analysis. Firstly, we conducted our analysis from the direct health service perspective only, we did not consider additional indirect (such as loss in economic productivity due to treatment related side effects) or intangible costs (e.g. loss in HRQoL). Secondly, perhaps of greater impact on the definitive cost-per-cure per patient, we have not taken into account the additional costs of retreating the 37.7 % of patients who failed PI treatment.

Analysis of cost-effectiveness is inevitably something that will change as prices of drugs, which make up the bulk of treatment costs, change. It is reasonable to expect that as there is more competition, prices will fall. Table 5 shows the effect of discount on cost-per-cure and given the high proportion of overall costs due to PI, discounts have a significant impact on cost-per-cure. However the results also show the proportional increase of non-discountable ‘other’ (largely AE management related) healthcare costs. This study provides valuable data to allow clinicians and healthcare decision makers to evaluate the real world total cost-per-cure amongst patients treated with current and future therapies. In particular, we provide a baseline dataset against which with the total cost per cure of emerging, all oral therapies can be compared and contrasted.

## Conclusion

The future healthcare burden of HCV in the UK, as in many countries, is significant. Further prospective real world safety, effectiveness and total cost data is needed for the new IFN free treatment combinations from different healthcare settings. Comparison with this data can provide evidence to inform future treatment decisions to ensure scarce healthcare resources are able to maximize patient outcomes.

## Additional file

Additional file 1: Supplementary material. (DOCX 39 kb)

## Abbreviations

AE: adverse event
BOC: boceprevir
HCC: hepatocellular carcinoma
HCV: hepatitis C virus
HRG: Health Resource Group
HRQoL: health-related quality of life
IFN: interferon
NHS: National Health Service
NICE: National Institute of Health and Care Excellence
PEG: pegylated interferon
PI: protease inhibitor
PR: pegylated interferon and ribavirin
RBV: ribavirin
SVR: sustained viral response
TVR: telaprevir
