# Supplementary material for: A new paradigm evaluating cost per cure of HCV infection in the UK
Source: Hepatol Med Policy. 2016 Apr 14;1:2. doi: 10.1186/s41124-016-0002-z (PMC5898515; doi:10.1186/s41124-016-0002-z)
Supplement: Supplementary file 1 — Supplementary material. (DOCX 39 kb) [file 41124_2016_2_MOESM1_ESM.docx]

**Table of contents**

[Table 1 Logistic regression: Probability of experiencing any AE 2](#_Toc423434572)

[Table 2 Repartition of AEs according to the association with treatment 2](#_Toc423434573)

[Table 3 List of 25 AEs classified as “Other” and assessed as “likely” related to treatment 3](#_Toc423434574)

[Table 4 Distribution of hospitalisations according to the association with treatment 3](#_Toc423434575)

[Table 5 Median cost per HCV cure - by outcome of prior therapy and by cirrhotic status 4](#_Toc423434576)

**Additional file** **1**

Table S1: Logistic regression: Probability of experiencing any AE

| **Parameter** | **OR** | **95% Wald Confidence Limits** | | **Standard Error** | **P value** |
| --- | --- | --- | --- | --- | --- |
| Treatment regimen (telaprevir vs boceprevir) | 0.46 | 0.22 | 0.98 | 0.19 | 0.04 |
| Age | 1.02 | 0.98 | 1.06 | 0.02 | 0.38 |
| Gender (male vs female) | 2.30 | 1.05 | 5.04 | 0.20 | 0.04 |
| Treatment status (treatment naïve vs experienced) | 0.47 | 0.22 | 0.99 | 0.19 | 0.05 |
| HIV co-infection (n=10)  (presence vs absence) | 1.62 | 0.42 | 6.27 | 0.35 | 0.49 |
| Liver disease  (No cirrhosis vs cirrhosis) | 3.13 | 1.19 | 8.22 | 0.25 | 0.02 |
| Depression (n=40)  (presence vs absence) | 0.67 | 0.30 | 1.46 | 0.20 | 0.31 |
| Diabetes (n=8)  (presence vs absence) | 1.04 | 0.20 | 5.33 | 0.42 | 0.97 |

Table S2 Repartition of AEs according to the association with treatment

|  | **“likely” (N)** | **“can’t tell” (N)** | **“unlikely” (N)** | **Total** |
| --- | --- | --- | --- | --- |
| Skin problem | 88 | 0 | 0 | 88 (49%) |
| Other* | 25 | 24 | 13 | 62 (35%) |
| Anaemia | 22 | 0 | 0 | 22 (12%) |
| Neutropenia | 6 | 0 | 0 | 6 (3%) |
| Total | 141 | 24 | 13 | 178 |

Table S3 List of 25 AEs classified as “Other” and assessed as “likely” related to treatment

| **Adverse Event Type** | **Frequency (N)** | **Percent (N/25)** |
| --- | --- | --- |
| Nausea | 7 | 28% |
| Mouth Ulcer | 2 | 8% |
| Oral Thrush | 2 | 8% |
| Sinusitis | 2 | 8% |
| Anal Irritation | 1 | 4% |
| Depression | 1 | 4% |
| Haemorrhoids | 1 | 4% |
| Injection wound site infection | 1 | 4% |
| Insomnia | 1 | 4% |
| Low Mood & Anxiety | 1 | 4% |
| Low Platelet Count | 1 | 4% |
| Oral Fungal Infection | 1 | 4% |
| Peri-Anal Itching | 1 | 4% |
| Severe Emotional Trauma and Paranoia | 1 | 4% |
| Small area of Red Rash plus isolated Scab | 1 | 4% |
| Thrombocytopenia | 1 | 4% |

Table S4 Distribution of hospitalisations according to the association with treatment

|  | **“likely” (N)** | **“can’t tell” (N)** | **“unlikely” (N)** | **Total** |
| --- | --- | --- | --- | --- |
| Anaemia [not outpatient blood transfusion] | 15 | 0 | 0 | 15 |
| Deep Vein Thrombosis | 0 | 0 | 2 | 2 |
| Diarrhoea and Vomiting | 0 | 2 | 0 | 2 |
| Magnesium Deficiency | 0 | 0 | 2 | 2 |
| Pancreatitis | 0 | 2 | 0 | 2 |
| AKI secondary to dehydration | 0 | 1 | 0 | 1 |
| Abdomen Pain - Biliary | 0 | 0 | 1 | 1 |
| Bell's Palsy | 0 | 1 | 0 | 1 |
| Campylobacter | 0 | 0 | 1 | 1 |
| Community Acquired Pneumonia | 0 | 1 | 0 | 1 |
| Headache | 1 | 0 | 0 | 1 |
| Hyperglycaemic | 0 | 0 | 1 | 1 |
| Pancytopenia, Neutropenic Sepsis and Anaemia | 0 | 0 | 0 | 1 |
| Rash | 0 | 0 | 0 | 1 |
| Renal Colic and Pyelonephritis | 0 | 0 | 1 | 1 |
| Sepsis | 0 | 1 | 0 | 1 |
| Shortness of Breath, Fever | 0 | 1 | 0 | 1 |
| T.A.C.E chemotherapy for HCC | 0 | 0 | 1 | 1 |
| Vomiting | 0 | 1 | 0 | 1 |
| Vomiting, Hypertension | 0 | 1 | 0 | 1 |
| Total | 16 | 11 | 9 | 36 |

Table S5 Median cost per HCV cure - by outcome of prior therapy and by cirrhotic status

|  | **Treatment naïve patients (n=74)** | **Prior relapsers (n=35)** | **Prior non-responders (n=36)** |
| --- | --- | --- | --- |
| **Total (n=145)*** |  | | |
| At 30% discount (% of base case) | £30,389 (80%) | £31,063 (79%) | £63,991 (76%) |
| At 40% discount (% of base case) | £27,141 (72%) | £28,374 (72%) | £57,312 (68%) |
| At 50% discount (% of base case) | £23,893 (63%) | £25,685 (66%) | £51,099 (61%) |
| **By liver disease stage** |  | | |
| ***Non cirrhotic (n=114)*** |  | | |
| At 30% discount (% of base case) | £28,566 (80%) | £29,021 (77%) | £56,315 (77%) |
| At 40% discount (% of base case) | £25,534 (72%) | £26,209 (70%) | £50,720 (69%) |
| At 50% discount (% of base case) | £22,503 (63%) | £23,397 (62%) | £45,128 (62%) |
| ***Cirrhotic (n=31)*** |  | | |
| At 30% discount (% of base case) | £49,799 (81%) | £38,946 (79%) | £80,596 (75%) |
| At 40% discount (% of base case) | £45,877 (75%) | £35,598 (73%) | £71,696 (67%) |
| At 50% discount (% of base case) | £41,958 (68%) | £32,249 (66%) | £62,800 (59%) |

*Prior status was not available for 9 patients
